# OAE: The Ontology of Adverse Events

**DOI:** 10.1186/2041-1480-5-29

**Published:** 2014-07-05

**Authors:** Yongqun He, Sirarat Sarntivijai, Yu Lin, Zuoshuang Xiang, Abra Guo, Shelley Zhang, Desikan Jagannathan, Luca Toldo, Cui Tao, Barry Smith

**Affiliations:** 1 University of Michigan, Ann Arbor, MI, USA; 2 US Food and Drug Administration, Silver Spring, MD, USA; 3 Merck KGaA, Darmstadt, Germany; 4 University at Texas Health Science Center at Houston, Houston, TX, USA; 5 University at Buffalo, Buffalo, NY, USA

**Keywords:** Ontology of Adverse Events, OAE, Adverse event, Ontology, Vaccine, Drug, Vaccine adverse event, VAERS, Drug adverse event, Design pattern

## Abstract

**Background:**

A medical intervention is a medical procedure or application intended to relieve or prevent illness or injury. Examples of medical interventions include vaccination and drug administration. After a medical intervention, adverse events (AEs) may occur which lie outside the intended consequences of the intervention. The representation and analysis of AEs are critical to the improvement of public health.

**Description:**

The Ontology of Adverse Events (OAE), previously named Adverse Event Ontology (AEO), is a community-driven ontology developed to standardize and integrate data relating to AEs arising subsequent to medical interventions, as well as to support computer-assisted reasoning. OAE has over 3,000 terms with unique identifiers, including terms imported from existing ontologies and more than 1,800 OAE-specific terms. In OAE, the term ‘adverse event’ denotes a pathological bodily process in a patient that occurs after a medical intervention. *Causal adverse events* are defined by OAE as those events that are causal consequences of a medical intervention. OAE represents various adverse events based on patient anatomic regions and clinical outcomes, including symptoms, signs, and abnormal processes. OAE has been used in the analysis of several different sorts of vaccine and drug adverse event data. For example, using the data extracted from the Vaccine Adverse Event Reporting System (VAERS), OAE was used to analyse vaccine adverse events associated with the administrations of different types of influenza vaccines. OAE has also been used to represent and classify the vaccine adverse events cited in package inserts of FDA-licensed human vaccines in the USA.

**Conclusion:**

OAE is a biomedical ontology that logically defines and classifies various adverse events occurring after medical interventions. OAE has successfully been applied in several adverse event studies. The OAE ontological framework provides a platform for systematic representation and analysis of adverse events and of the factors (*e.g.*, vaccinee age) important for determining their clinical outcomes.

## Background

A medical intervention is a medical procedure or application intended to relieve or prevent illness or injury. The medical intervention can be an administration of a drug, a vaccine, a special nutritional product (for example, a medical food supplement), or it can be the use of a medical device. In the wake of a medical intervention, adverse events (AEs) may occur which lie outside the intended consequences of the intervention. These AEs are pathological bodily processes [1]. Severe AEs include triggering of Guillain-Barre or Stevens-Johnson Syndrome paralysis and, in extreme cases, death. Such AEs may result in hospitalization of the patient and requiring special care. Although typically having low incidence rates, they may impact the usage or regulation of vaccine, drug, or medical devices in the market. To monitor and investigate adverse events of various types, reporting systems have been established to collect the relevant information. For example, the USA national vaccine safety surveillance programs include the Vaccine Adverse Events Reporting System (VAERS) [2] and the Food and Drug Administration (FDA) Adverse Events Reporting System (FAERS) [3], established, respectively, for the spontaneous reporting of vaccine and of drug-associated AEs.

To improve representation and organization of adverse event information, efforts have been undertaken over the years to develop different vocabulary resources, including the Medical Dictionary for Regulatory Activities (MedDRA) [4], the Common Terminology Criteria for Adverse Events (CTCAE) [5], and the World Health Organization (WHO)’s Adverse Reaction Terminology (WHO-ART) [6]. MedDRA is an adverse event coding vocabulary preferred by the FDA and utilized by VAERS and FAERS, as well as many clinical trials. CTCAE, a product of the USA National Cancer Institute (NCI), is a standardised vocabulary used in assessing AEs associated with drugs for cancer therapy. WHO-ART is a dictionary maintained by the WHO to serve as a basis for rational coding of adverse reaction terms.

While these resources have played a central role in standardizing and improving AE vocabulary use worldwide, their lack of text definitions and logical classification hierarchies poses problems for automatic search and retrieval and for computational analysis and aggregation [7]. The Ontology for Adverse Events (OAE) is designed to address these issues by providing logically well-formed definitions and an associated structured classification. These definitions and classification function as the “abstraction” of the information from highly specific “particular” (or “instance”) adverse events to more general “universals” (or “classes”) that show commonalities often not obvious from individual data. As first illustrated in [8] and discussed also below, the application of OAE appears to support reasonable classification and analysis of the vaccine adverse events (VAE) reported in the clinical VAE case report system. MedDRA and other classical systems focus on the representation of the symptoms or diseases that are the adverse event outcomes of clinical findings. They, thus, do not take into account other elements (e.g., patient age) of the process that leads from initial medical intervention to subsequent outcomes. The OAE is designed to serve as a complementary resource that will fill this gap of treatment-clinical observation association by providing a means of linking the content coded by these systems to other relevant biological and clinical information.

Biomedical ontologies are consensus-based controlled vocabularies of entities and relations modelling a part of the biomedical world, which are represented in both computer and human interpretable forms. They thus go further in providing support for computational analysis of data than the existing vocabulary resources. The Adverse Event Ontology (AEO) was initially developed by transferring those ontology terms representing vaccine adverse events from the Vaccine Ontology (VO) [9,10]. The top level AEO adverse event representation was also partially based on the work conducted in the European ReMINE project [11]. In our previous AEO paper [12], we defined the term ‘adverse event’ as: a pathological bodily process that is induced by a medical intervention. This definition in the previous version of the ontology of AEs assumed a causal association between an adverse event and a medical intervention. A problem with this definition is that it does not align with the common usage of the term ‘adverse event’ in medical, pharmacological and public health contexts, where it is generally impractical to distinguish the causal adverse consequences from all the bodily processes that unfold in a patient temporally subsequent to a given medical intervention. The FAERS and VAERS systems thus state explicitly that they make no assumption of a causal relation between an adverse event and a medical intervention. The assumption of causality in our preceding ontology would imply too large a gap between the ontology and actual practice. Above all, this assumption would make it difficult to use the term ‘adverse event’ to represent individual cases, since the existence of a causal relation is in many cases hard to verify. Furthermore, due to a name conflict with the “Anatomical Entity Ontology” that has the same abbreviation “AEO”, our Adverse Event Ontology (AEO) was renamed the Ontology of Adverse Events (OAE) in the Fall of 2011. In OAE, ‘adverse event’ (OAE_0000001) is defined as to assume no causal association, while those adverse events for which there is a causal association with an intervention are defined as a subclass of ‘adverse event’ and named as ‘causal adverse event’ (OAE_0000003). The latter term is to be used only when there is definitive evidence (including biological and statistical evidences) to assert such a causal association under specified conditions. We contend that with this change the OAE becomes more robust as a representation of the domain of adverse event reporting.

In addition, other updates have been made to the OAE as compared to the original AEO. A large number of new OAE terms derived from a number of use cases have been added. Different ways of representing and analyzing the causal association between AEs and medical interventions have been classified and represented in OAE, and the ontology has also been used in several studies, which will be introduced in this paper.

### Breadth and Scope

The OAE ontology is a community-based biomedical ontology in the domain of adverse events. OAE clearly differentiates *adverse event* and *causal adverse event*, with the latter a subtype of the former. A major effort in OAE is to represent ontologically various AEs on anatomic locations and adverse outcomes (including symptoms, signs, and processes). OAE includes many logic definitions formulated by using terms from existing ontologies (for example the UBERON anatomy ontology). This strategy links OAE with established ontologies and supports computer-assisted integration and reasoning. Since OAE defines an adverse event as a process subsequent to a medical intervention, the ontology provides a logical first step in the representation of this whole process. Such ontological definition allows the development and application of new analysis methods to better understanding the mechanisms of adverse events associated with or induced by different medical interventions. OAE also provides a framework for recording and analyzing the associations recorded on product labels for example between vaccine or drug administration and medically relevant events. The scope of OAE is very specific and should not be confused with other relevant ontologies. OAE does not target adverse event reporting by following the pattern of the existing Adverse Event Reporting Ontology (AERO) which focuses on the ontological representation of the vaccine AE data or information using the Brighton vaccine AE definitions [13]. Unlike OAE, AERO does not define ‘adverse event’ as a pathological bodily process. By following the principle of ontological realism we argue that the representation of data should be built as far as possible on the real-world entities to which such data relate. Finally, OAE is not an ontology of symptoms or signs as the indications of illness or diseases. The appearance of various symptoms or signs (*e.g.*, fever) is rather an *outcome* of an adverse event, thus denoted by the suffix “AE” (*e.g.*, a fever adverse event or “fever AE”).

### Authority and provenance of OAE

OAE targets two communities: the adverse event community and the OBO Foundry ontology community. As concerns the former, we have focused our ontology development on two important research communities, targeting vaccine adverse events and drug adverse events, respectively, and our team includes experts in both of these areas. For example, Dr. Yongqun He (co-author) is a domain expert in vaccinology, vaccine adverse events, and ontology development [8,14]. Dr. Luca Toldo (co-author) is an expert in drug adverse events [15-17]. Expanding the OAE ontological analysis of VAERS data [8], Dr. Sirarat Sarntivijai is now expanding and applying OAE and ontology knowledge mapping to represent and analyze drug-associated AEs in her systems pharmacology research. Her OAE research has obtained strong support and collaboration from clinical experts at the FDA’ Office of Clinical Pharmacology. As an ontology in the OBO Foundry ontology library, the development of OAE follows the OBO Foundry principles [18]. Dr. Barry Smith (co-author) is the founder of the Basic Formal Ontology (BFO) and also the one of the founders of the OBO Foundry. Our core development team has also included experts in semantics web (Dr. Cui Tao), medical informatics (Yu Lin, MD, PhD), software developer (Zuoshuang Xiang), and many students. Our developmental effort has received technical supports from both the adverse event community and the OBO Foundry ontology community, as demonstrated by positive feedbacks we received from three recent international adverse event related workshops [19-21].

As described above, the OAE was originally derived from the vaccine adverse event branch of the Vaccine Ontology (VO) [9,10] and from the European ReMINE project [11]. New OAE adverse event terms have been generated on the basis of clinical adverse event reports in the VAERS [22] and the FDA [3]. We have referenced MedDRA in our OAE development by cross-referencing related MedDRA identifiers. The data models of adverse event analysis provided by the Clinical Data Interchange Consortium (CDISC) [23] were also referenced. Peer-reviewed journal articles have been used wherever possible as references for adverse event terms included in OAE.

### Construction and content

#### OAE development methods and statistics

The development of OAE follows the OBO Foundry principles, including openness, collaboration, and use of a common shared syntax [18]. We also adhere to the principles of ontological realism [24]. OAE is aligned with the Basic Formal Ontology (BFO) (http://www.ifomis.org/bfo) version 2.0.

The latest OAE is available for public view and download at http://sourceforge.net/projects/oae/. The Web Ontology Language (OWL) is used as representation language and the ontology is edited using the Protégé 4 Ontology Editor (http://protege.stanford.edu). For adverse event-specific terms, new identifiers with the “OAE_” prefix plus seven-digit auto-incremental numbers were generated. OntoFox (http://ontofox.hegroup.org/) [25] was used to extract ontology terms from external ontologies and import them into the OAE where needed.

As of April 20, 2014, OAE has 3,086 terms (or called representational units) (Table 1). To support ontology term reuse and interoperability, OAE imports terms that are available in established ontologies. The terms from existing ontologies are imported in two different ways: one is to import the whole ontology (here BFO and RO); the other is to import individual terms from existing ontologies using OntoFox [25]. In accordance with the OBO Foundry Principles, we need to share development effort with other ontology initiatives. It is noted that imported terms keep their original IDs. By using the OntoFox software [25], the relations between imported entity terms and the entity properties are also retained. The OAE-specific terms include 1,834 classes and three object properties (Table 1). Compared to the 484 ontology terms included in the last AEO publication in 2011 [12], 2,602 new terms (over fivefold more terms) have been added to OAE, representing significant progress of the ontology.

**Table 1:** Summary of ontology terms in OAE or imported from existing ontologies as of April 20, 2014

**Ontology names**	**Classes**	**Object properties**	**Datatype properties**	**Annotation properties**	**Total**
OAE	1830	4	0	0	1834
BFO (Basic Formal Ontology)	37	83	0	2	122
BSPO (Spatial Ontology)	0	13	0	0	13
DOID (Disease Ontology)	1	0	0	0	1
IAO (Information Artifact Ontology)	8	1	0	16	25
OBI (Ontology for Biomedical Investigations)	11	7	0	2	20
OGMS (Ontology for General Medical Science)	4	0	0	0	4
PATO (Phenotypic Quality Ontology)	0	0	0	3	3
RO (Relation Ontology)	0	20	0	1	21
UBERON (Uber Anatomy Ontology)	883	0	0	0	883
VO (Vaccine Ontology)	7	0	0	0	7
Other ontologies*	27	56	1	70	241
**Total**	2808	183	1	94	3,088

Note: *the name and statistics of other ontologies used in OAE can be found on the Ontobee website: http://www.ontobee.org/ontostat.php?ontology=OAE.

#### OAE design patterns of adverse events and causal adverse events

Basic Formal Ontology (BFO; http://www.ifomis.org/bfo) is a small, upper level ontology provides a common ontological architecture for a series of domain ontologies at different levels of granularity [24,26]. BFO has been used by more than 100 ontologies belonging to scientific and other domains and the alignment with BFO makes OAE compatible with other BFO-based ontologies in the biomedical field. BFO includes two branches: the continuant branch, representing types of entities that persist through time while preserving their identity; and the occurrent branch, representing entities (such as processes and the temporal regions they occupy) that have temporal parts and unfold or develop through time.

In OAE, the term ‘adverse event’ is defined as: a pathological bodily process (OGMS_0000060) that occurs after a medical intervention. The term ‘pathological bodily process’ is taken from the Ontology for General Medical Science (OGMS), where it represents a bodily process that is clinically abnormal [1]. OGMS, too, is a BFO-aligned ontology. OGMS: pathological bodily process is a subtype of BFO:process. The ‘medical intervention’ is a planned process (also a subtype of BFO:process) that has the goal of diagnosing, preventing or relieving illness or injury. We originally developed this term in OAE and have proposed to transfer this term to OGMS.

**Figure 1:**
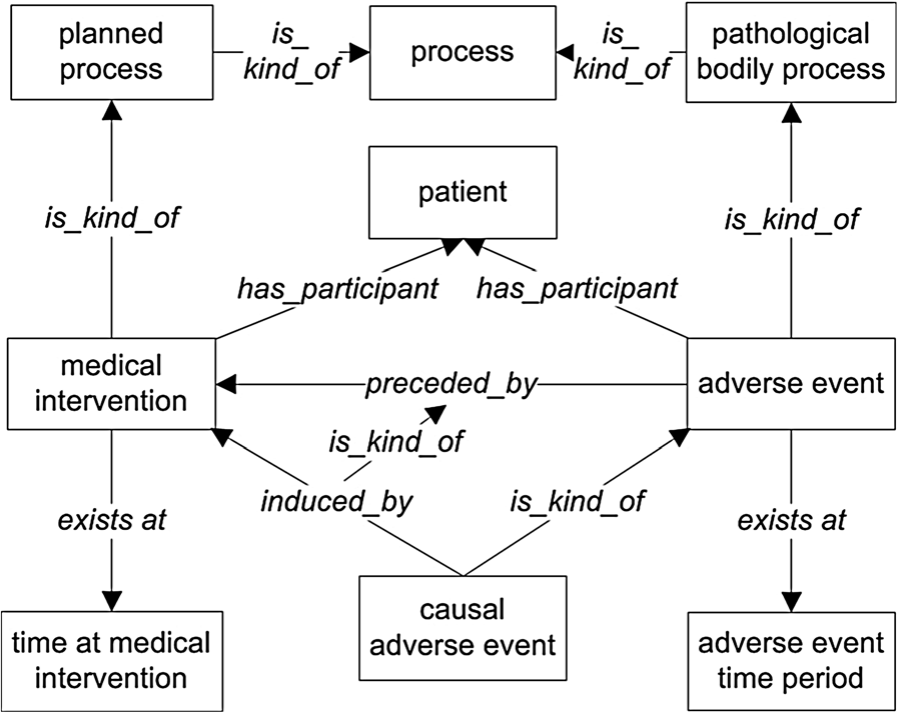
**Basic design pattern of OAE ‘adverse event’ and ‘causal adverse event’.** All terms inside boxes are ontology classes, and the other terms are ontology relations. The is-kind-of (*i.e.*, rdfs:subClassOf) relations are highlighted with bold font. The other relations come from OAE or other existing ontologies. The detailed information of the class and relation terms used in this figure is available in Additional file 1: Table S1 and Additional file 2: Table S2.

An adverse event is part of a process that starts when a medical intervention is conducted and involves some observation event in which a clinical outcome is observed or detected (Figure 1). Note that Additional file 1: Table S1 and Additional file 2: Table S2 provide detailed information about the classes and relations used in all the figures presented in this manuscript. In simplified terms we have the following elements:

(1) p1: a medical intervention (for example, an act of vaccination);

(2) pa: a patient;

(3) t1: the time at which the medical intervention occurs;

(4) p2: a process leading to a clinically abnormal outcome;

(5) t2: the time at which the clinically abnormal outcome (e.g., fever) is first manifested.

Both *medical intervention* (p1) and *adverse event* (p2) are subclasses of BFO *process* with instances occurring at specific BFO:*temporal regions*. The difference between p1 and p2 is that medical intervention is a process that is planned by some human being (thus it is an OBI:planned process) and implemented on a patient (pa), while an adverse event is an unplanned (unintended) event that is subsequent thereto. The temporal region at which the medical intervention occurs in a given patient is always earlier than the temporal region at which the adverse event occurs: t1 *precedes* t2. Therefore, the adverse event p1 is *preceded by* the medical intervention p2. It is noted that the clinical abnormal outcomes may have different types, including symptom (for example, rash), sign (for example, white blood cell count decreased), or process (for example, bacterial infection).

**Figure 2:**
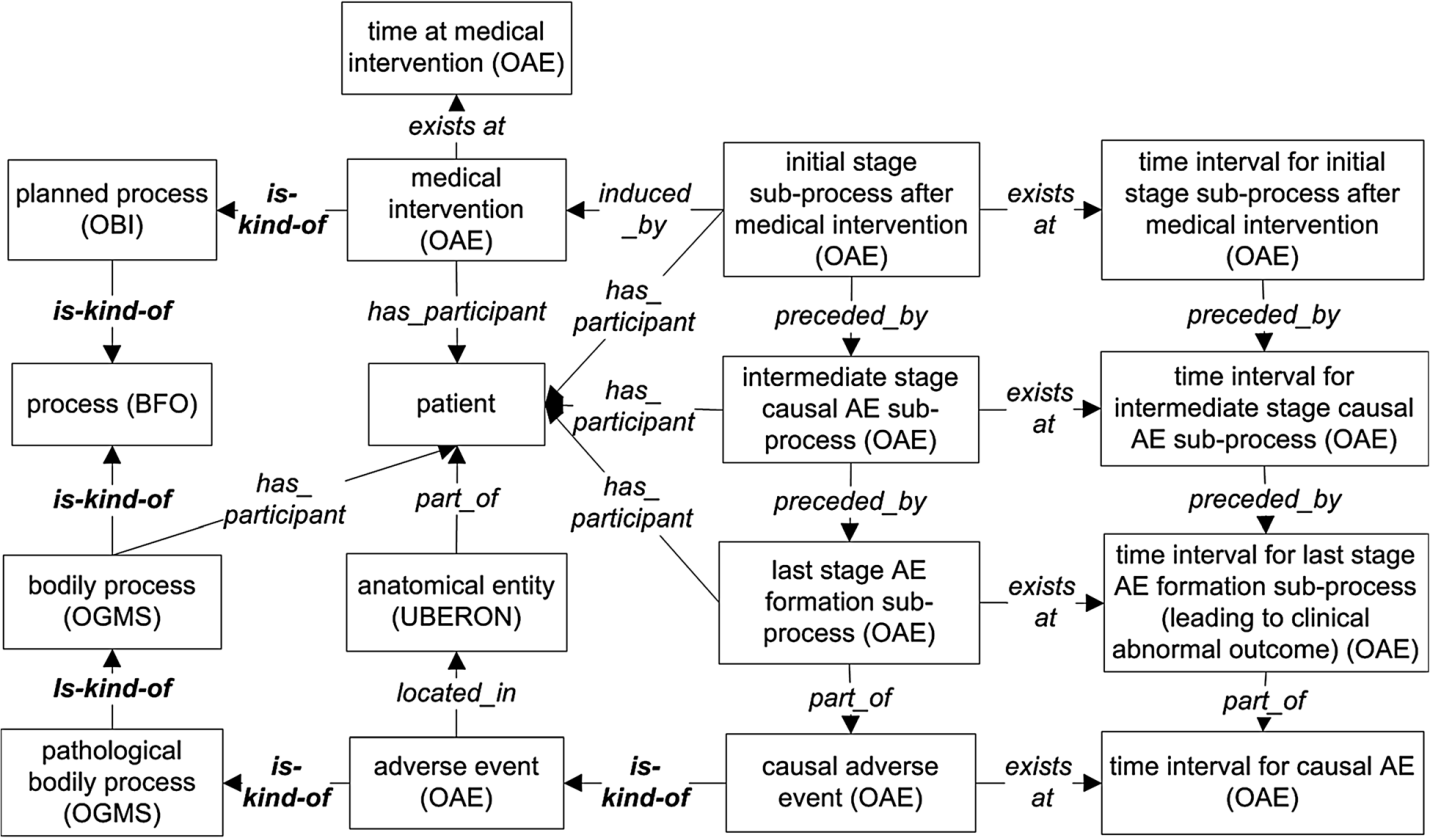
**OAE causal adverse event design pattern.** All terms inside boxes are ontology classes, and the other terms are ontology relations. The is-kind-of relations are highlighted with bold font. The other relations come from OAE or other existing ontologies. The detailed information of the class and relation terms used in this figure is available in Additional file 1: Table S1 and Additional file 2: Table S2.

Both ‘adverse event’ and ‘causal adverse event’ are used in relation to events occurring after a medical intervention. The major difference between them is that the latter is used if and only if it has been established that the event in question occurred as a result of (was caused by) this intervention. Thus the term ‘causal adverse event’ represents the existence of data of a certain sort, namely data establishing a causal association between these two processes (Figures 1 and 2). The causal relation is represented by the object property term ‘induced_by*’* (occurs as a causal consequence of) rather than being annotated by the relation ‘preceded by’ in the occurrence of any AE. The relation ‘induced_by’ (used to define ‘causal adverse event’) is a special case of ‘preceded by’ (used to define ‘adverse event’). For the logical definition of OAE ‘causal adverse event’ , the expression ‘induced_by’ is used instead of ‘caused_by’ to justify and emphasize the fact that the causality linking an adverse event to a medical intervention is indirect and connected by multiple subprocesses in a causal chain as detailed below.

In OAE, the term ‘causal adverse event’ is fully defined with an equivalence class axiom as: *‘adverse event’ and (‘induced_by’ some ‘medical intervention’),* but the term ‘adverse event’ is not. This means that a ‘causal adverse event’ can be recognized by an ontology reasoner but an ‘adverse event’ cannot. The reason is that there are other criteria that have not been formalized to be reasoned for being an adverse event other than merely an incident that follows some medical intervention. For example, the temporal association where a fever occurs one year after receiving an influenza vaccination cannot be drawn as it is most likely that this fever is not an adverse event following that vaccination. It is difficult to infer that a clinical outcome is an adverse event by a temporal association alone.

Due to insufficient data or technology limitation, some potential real ‘causal adverse event’ cannot be asserted. Events of this sort are indistinguishable from non-causal adverse events and will thus be annotated simply using ‘adverse event’.

### Further OAE modelling of sub-processes in a causal adverse event chain

In the above section, we used “p2” to represent the adverse event process leading to a clinically abnormal outcome. While p2 adverse event process always occurs, such a process may not be triggered by the medical intervention (“p1”). When p2 is indeed triggered by p1, we call such an adverse event ‘causal adverse event’. In this section, we will study how a medical intervention can lead to the clinically abnormal outcome in the case of a causal adverse event.

Triggered by a medical intervention, we argue that a causal adverse event process is indeed a chain composed of a series of sub-processes. Specifically, a medical intervention initiates a series of processes in which several independent continuants (for example, anatomical parts of the body of the patient) participate in a variety of ways. Two neighboring processes in the series of processes share at least one continuant (*e.g.*, the same anatomic part) among their participants. Other processes need not share any continuant in this way. As shown in Figure 2, OAE separates the causal chain of an adverse event process into three subtypes of processes:

(i) *Initial stage causal AE sub-process:* This stage happens immediately after medical intervention, and the stage ends when the single chain process begins to be forked (or separated) into different sub-processes, some leading to positive preventative or therapeutic effect, some leading to noises, and some leading to adverse events.

(ii) *Intermediate stage causal AE sub-process:* One of the forked sub-processes will be developed further in a temporal fashion and may include a series of intermediate smaller sub-processes, one of which will lead to the last final stage as described below.

(iii) *Late stage AE formation sub-process:* This last stage is the *execution* stage leading to pathological clinical outcome (including the *appearance* of the outcome). This stage is similar to the caspase cascade as the execution stage of apoptotic cell death [27].

To illustrate the whole process, here we describe an example of how the administration of an influenza vaccine induces fever in human. After the vaccination process, the vaccine comes to the bloodstream, attracts a large number of immune cells, and triggers initial immune responses. This stage is considered as an ‘initial stage sub-process after medical intervention’. After the shared initial stage triggered by the medical intervention, different intermediate stage sub-processes will occur. Some of these sub-processes will lead to a positive (intended) outcome, *i.e.*, adaptive immunity against infectious influenza virus infection. Some sub-processes will lead to negative (adverse) outcomes, *i.e.*, various clinical abnormal outcomes. More than one abnormal outcome may occur. Some “noise” sub-processes leading to no positive or negative outcomes may also occur. In the end of this stage, those noise sub-processes will disappear. This stage includes one ‘intermediate stage causal AE sub-process’ that will result in the synthesis of the prostaglandin E2 (PGE2). The release of PGE2 will stimulate the hypothalamus to increase body temperature, leading to the appearance of fever [14]. The process from the release of PGE2 to the fever outcome is the execution stage of the fever outcome; therefore, it is regarded as the ‘late stage AE formation sub-process” that directly results in the pathological clinical outcome.

### Representation of factors influencing adverse events

**Figure 3:**
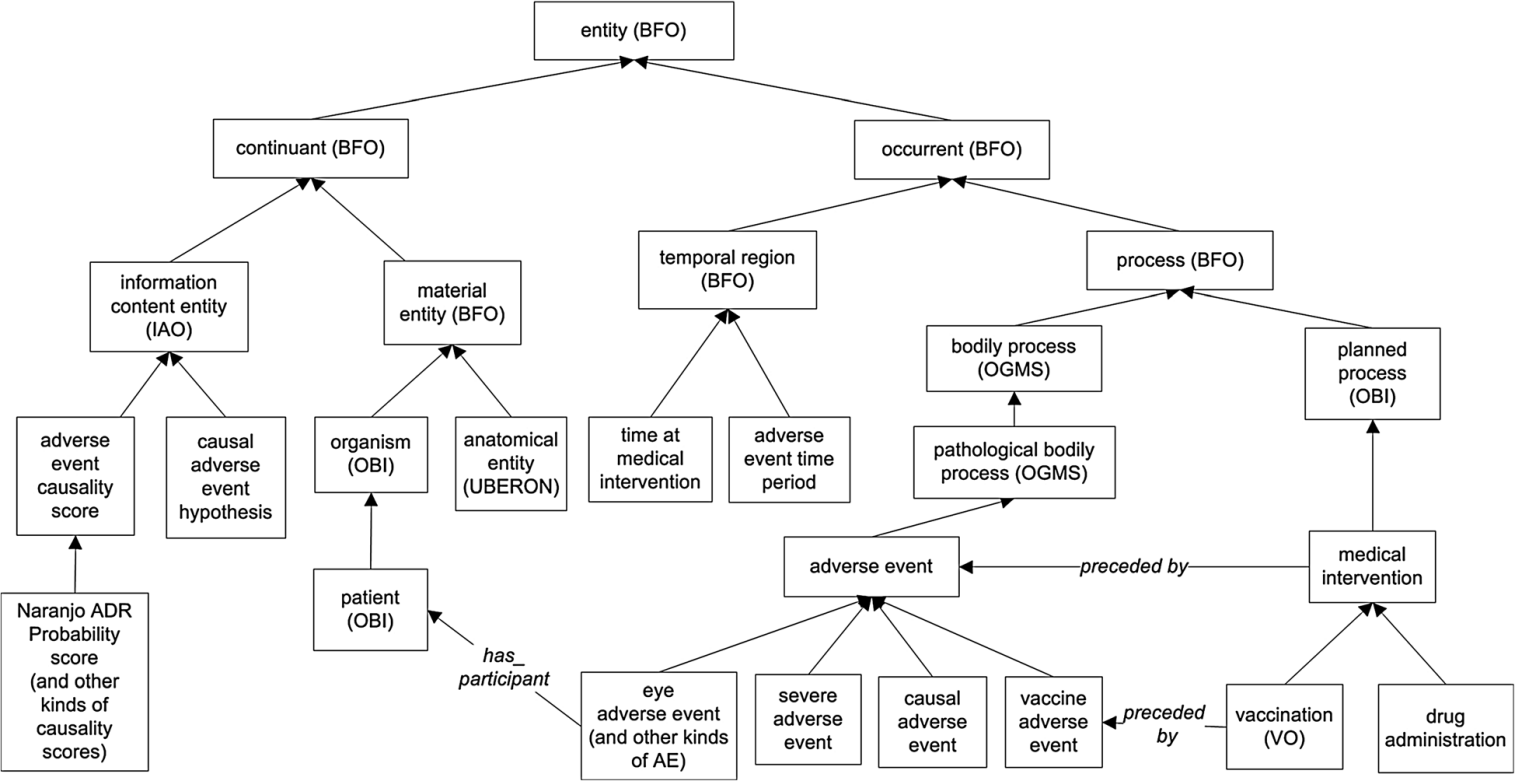
**Key ontology terms in OAE.** Except those with special labels, all arrows represent the same *is-kind-of* relations. Except those terms labelled with ontology abbreviation names, all terms inside boxes come from OAE. The detailed information of the class and relation terms used in this figure is available in Additional file 1: Table S1 and Additional file 2: Table S2.

OAE provides a framework that allows the representation and analysis of different factors and mechanisms that possibly influence adverse event outcomes. For example, two major factors that determine distinct AEs associated with killed and live attenuated influenza vaccines include the viability of vaccine organism and the vaccination route [8,10]. A killed influenza vaccine is administered through intramuscular injection. A live attenuated influenza vaccine is administered through intranasal spray. While it is still unclear whether the vaccination route affects the adverse event outcomes [10], our linkage between adverse events and vaccination routes provides us a way to systematically study this issue. Since OAE links the patients to the adverse event (Figure 2), it is possible to link the patient records (*e.g.*, age, gender, vaccine administered, and vaccination method) to adverse events. An example is that vaccinees in different age groups likely have different occurrence rates for specific AEs. Such age-AE associations, clearly documented in vaccine package inserts, have been ontologically recorded in our recent OAE-based study [28]. Therefore, the OAE framework provides the computational infrastructure to model the relations between different factors and the clinical outcome.Genetic background of a patient also affects the occurrence of the adverse event in this example. Knowledge of intricate drug-patient and drug-drug interactions is also crucial to determining final adverse drug event outcomes. Some adverse events happen due to cross-interactions between drug and food (as for example in the case of statins and grapefruit). A patient may have taken a medicine or retain a pre-existing condition (e.g., drinking alcohol). The alcohol may have an increased effect on some adverse event induced by a drug (e.g., benzodiazepines). Through the inclusion of a patient with specific conditions in the OAE design pattern (Figure 3), OAE outlines the computational infrastructure with the capability to capture these potential interaction mechanisms through ontological linkages with the long-term goal of understanding the fundamental basis of these specialized causal events. When new evidences become known to support the likelihood of a drug-AE causal association, appropriate measures can be rationally designed to counter these adverse effects.

### OAE adverse event hierarchy

Figure 3 lists many key ontology terms and their hierarchical relations in OAE. As described above, the OAE adverse event is a subtype of OGMS ‘pathological bodily process’ , which is a subtype of BFO:process. Different types (subclasses) of adverse events exist and can be classified on the basis of the type of medical intervention (*e.g.*, vaccine adverse event), anatomy (*e.g.*, eye adverse event), severity (*e.g.*, severe adverse event), or causality (*e.g.*, causal adverse event). The ‘medical intervention’ is an OBI ‘planned process’ , another subtype of BFO:process. Besides vaccination and drug administration, other medical interventions include surgery process, medical nutritional product usage, and medical device usage (not shown in Figure 3). Each of the interventions has a corresponding adverse event type (*e.g.*, vaccine adverse event). Different adverse event-related time instances or periods are defined under BFO ‘temporal region’. Both process and temporal region are types of BFO ‘occurrent’ (Figure 3).

OAE imports many UBERON anatomy terms [29] for logically defining the anatomic regions of those adverse events classified by anatomy. UBERON is an integrated cross-species ontology representing a variety of anatomical entities [29]. The Foundational Model of Anatomy (FMA) [30] is domain ontology of the concepts and relationships pertaining to the structural organization of the human body. OAE uses species-neutral UBERON instead of human-specific FMA for anatomic region representation because OAE is also applicable for veterinary animal use. A patient is an organism that has the role of a ‘patient role’. The species of patients are specified by the NCBI Taxonomy ontology [31]. Both anatomic entity and organism are types of BFO ‘material entity’. The material entity and different types of information content entity are subclasses of BFO:continuant (Figure 3).

**Figure 4:**
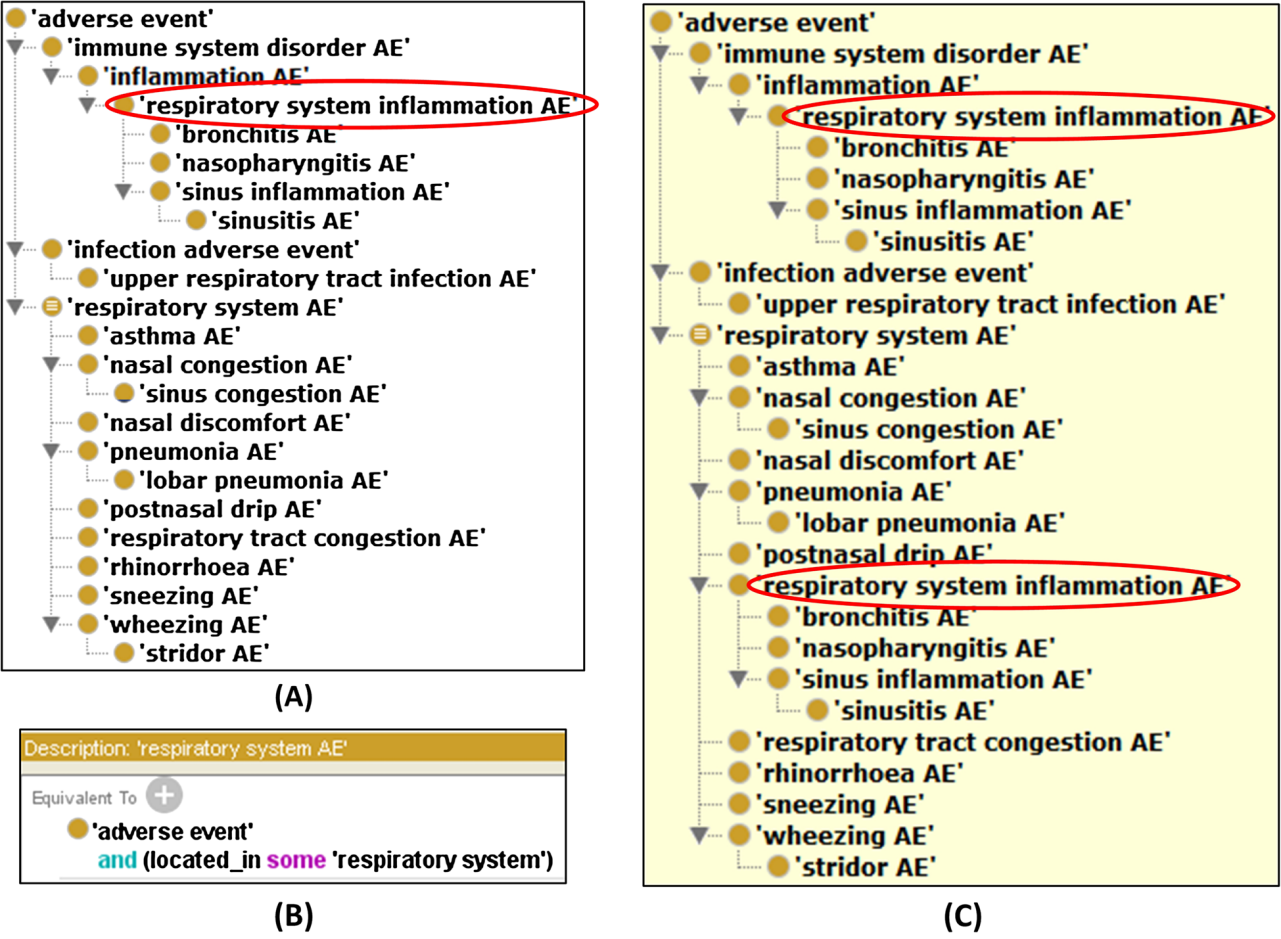
**OAE classification of FluMist-associated adverse events (AEs). (A)** Representation of those FluMist-associated adverse events (leave nodes) and their asserted hierarchy. **(B)** An OAE axiom defining equivalent class ‘respiratory system AE’. **(C)** Inferred hierarchy after reasoning using the HermiT reasoner available as a plugin in the Protégé-OWL editor in a Windows-7 computer. After reasoning, ‘respiratory system inflammation AE’ is classified as ‘respiratory system AE’. By comparing the places of the term ‘respiratory system inflammation AE’ (highlighted with red oval box) in **(A)** and **(C)**, this term appears in both places in the inferred version in **(C)**. This has not been moved, but an additional parent class has been added. It is noted that the execution of the reasoning was finished within seconds.

It is noted that the primary goal of current OAE development is not to provide a comprehensive solution to the causality problem. Instead, OAE merely specifies certain features of causal adverse events in order to provide a first step towards a better understanding. It is typically unlikely to assert an AE causality as a definite “yes” or “no” conclusion. Instead, the AE causality is usually defined as a probability or hypothesis. OAE has now incorporated terms relating to a number of established methods used for AE causality analysis. For example, it includes terms such as ‘Naranjo ADR Probability score’ that can be used to annotate data concerning the likelihood of a causal adverse drug reaction (ADR) based on a patient’s answers to a list of pre-designed questions [32]. The CDISC system [23] distinguishes five causality levels, including: (1) not related, (2) unlikely related, (3) possibly related, (4) probably related, and (5) definitely related. These types of causality have been represented in OAE as well. In addition, AE case reports in a case reporting system such as VAERS can be analysed statistically using methods such as filtering based on a case number cutoff, proportional reporting ratio (PRR) [33], and Chi-square test, in order to screen for possible causal AEs [8]. To make more convincing causality assessment using a statistical method, randomized, well-controlled experimental design for evaluating a causality hypothesis is typically required [34,35]. These statistical methods can be used to address any possible ‘causal adverse event hypothesis’. As exemplified in Figure 3, terms relating to such methods have been included in OAE. The various mechanisms for causality assessment represented in OAE provide different options for researchers and tool developers to assess the occurrence and causes of adverse events.The AE hierarchy demonstrates the general organization of various AE terms in OAE. OAE defines many AE terms based on medical intervention such as ‘drug adverse event’ , and ‘vaccine adverse event’. However, these terms are high level terms, and OAE does not intend to represent specific adverse events associated with each vaccine, drug, or surgery. The majority of AE terms in OAE, such as pneumonia AE and dry throat AE, are classified based on clinical outcomes (including symptoms, signs, and abnormal processes) and anatomic entities. Many terms may fit under two or more parent AE terms. For example, a ‘respiratory system inflammation AE’ may be asserted under ‘respiratory system AE’ or ‘inflammation AE’. OAE avoids the use of multiple inheritance. Our general strategy is that when both a clinical outcome-based AE term (such as ‘inflammation AE’) and an anatomy-based AE term (such as ‘respiratory system AE’) are candidates for being included as asserted terms in OAE, then we assert the clinical outcome-based term (here: ‘inflammation AE’), and allow the other parent term to be obtained by reasoning. In this example, we assert the term ‘respiratory system inflammation AE’ under ‘inflammation AE’ , and through reasoning (see the next section), it is inferred as a child term of ‘respiratory system AE’ (Figure 4). The reason of this choice is that the clinical outcome is often more critical to the physicians and the location can be easily defined using the UBERON anatomy ontology.

### Usage

Current development of OAE has emphasized the representation and analysis of adverse events associated with vaccine and drug administrations. Several papers have been published in terms of the application of OAE in vaccine adverse event studies [8,14,28,36,37]. Two studies have been undergoing on using OAE in analysis of drug adverse events. For better understanding of the features and usage of OAE, these studies are introduced below.

### OAE-based vaccine adverse event studies

#### (1) Analysis of clinical vaccine adverse event case report data

The unsystematic reported vaccine AE cases in the VAERS system contain both coincidental events and those truly caused by vaccines. The data stored in such a reporting system can be used to generate hypotheses about potential causal association between adverse events and medical interventions. Such a hypothesis, represented by the OAE term *causal adverse event hypothesis*, becomes very important for attempts to improve the public health when a dramatically large amount of cases are reported following the same medical intervention.

To analyse reported adverse event cases, an OAE-based AE analysis algorithm called Combinatorial, Ontology-based Detection of AE (CODAE) was developed [8]. CODAE first extracts the VAERS VAE case report data annotated by MedDRA. A set of well-recognized statistical methods (containing filtering, Proportional Reporting Ratio (PRR) [33], and Chi-square test) were then used to identify statistically significant AEs associated with one or one group of vaccines, *e.g.*, live attenuated influenza vaccine FluMist. It is noted that these statistical methods (*e.g.*, Chi-square test) have been represented in the current version of OAE. The usage of these statistic methods resulted in the identification of a list of “enriched” AE terms that are statistically significant compared to the background noise recognized by all the AE case reports for all vaccines collected in VAERS. Through MedDRA-OAE term mapping, the statistically enriched AE terms can then be classified and analysed using the OAE hierarchical structure [8].

The CODAE strategy was used to analyse and compare the AEs associated with two types of influenza vaccines: trivalent (killed) inactivated influenza vaccine (TIV) and trivalent live attenuated influenza vaccine (LAIV). In the USA, each year an average of 20,000 children under the age of 5 are hospitalized because of influenza complications (http://www.cdc.gov/flu/protect/children.htm). The single best approach to protecting oneself against seasonal flu and its potentially severe complications is to receive a seasonal influenza vaccine each year. However, a seasonal influenza vaccine may also cause adverse side effects. Using all possible data in the VAERS, our CODAE statistical analysis identified 48 TIV-enriched and 68 LAIV-enriched AEs (PRR > 2, Chi-square score >4, and the number of cases >0.2% of total reports). The OAE-based study on this CODAE AE classification method found that TIV was more likely to be associated with neurological and muscular abnormal processes such as paralysis, movement disorders, and muscular weakness. In contrast, LAIV-enriched AEs included inflammatory response and respiratory system disorders. We also found that, although rare, two severe adverse events (Guillain-Barre Syndrome and paralysis) were more likely to be present in TIV-vaccinated patients [8]. Such AE group enrichment results were difficult to obtain without the support of OAE.

To demonstrate how OAE-based vaccine enrichment works, we extracted all 14 respiratory system adverse event terms associated with FluMist, the only LAIV vaccine in the market, from the CODAE analysis of influenza vaccine AEs [8]. These terms and other related terms were extracted using OntoFox [25] and visualized using the Protégé OWL editor [38] (Figure 4). Different inflammatory AEs in various anatomic regions (*e.g.*, respiratory system inflammatory AEs) have been asserted under inflammatory AE (Figure 4A). A *located_in* relation can be used to logically define an anatomic region as the location of a clinically abnormal process, for example, *located_in some respiratory system*. After reasoning using the HermiT OWL ontology reasoner (http://hermit-reasoner.com/), the term ‘respiratory system inflammatory AE’ was automatically inferred as a child of ‘respiratory system AE’ (Figure 4C). The inferred hierarchy can then be used for AE group enrichment analyses.

The VAERS database includes unstructured case narratives that are not well-organized and used. The unstructured narratives usually contain rich data about the temporal features of the AEs. In another study, the OAE was leveraged to model the temporal relations of post-vaccination events [36]. The unstructured nature of the narrative data, makes automatic processing difficult. In the reported study [36], OAE, VO and the Clinical Narrative Temporal Relation Ontology (CNTRO) [39] were used to represent data in VAERS narratives in a “machine-understandable” way, so that the data can be easily queried and further analyzed. The usage of OAE makes it possible to classify the extracted AE results. A VAERS case report was presented as a use case for the ontological representations. The advantages of using the ontology-based semantic web representation and data analysis were emphasized [36]. It is noted that in order to more specifically measure how OAE can be used for improving AE classification, more statistical studies (e.g., analysis of precision and recall) will need to be conducted in the future.

#### (2) Classification and analysis of vaccine adverse event report in licensed vaccine package insert documents

The USA FDA website contains vaccine package insert documents for commercial human vaccines currently licensed in the USA [40]. The difference between this study and the VAERS study introduced above is that in contrast to the unsystematic treatment of clinical data by VAE reports stored in VAERS, VAEs resting on licensed vaccine package insert documents provide results from randomized, well-controlled clinical trials. Therefore, the adverse events recorded in the official vaccine package inserts are known vaccine-specific adverse events existing in vaccinated populations.

To better analyse the data pertaining to known vaccine adverse events, an Ontology of Vaccine Adverse Events (OVAE) was recently developed as an OAE extension [28]. OVAE was first used to represent the vaccine adverse events reported in the FDA vaccine package insert documents. OVAE imports from OAE terms referring to 87 distinct types of adverse events, which have been associated with 63 human vaccines licensed in the USA. OVAE also imports information relating specific licensed human vaccines taken from the Vaccine Ontology (VO). By importing terms from OAE and VO, OVAE is able to represent vaccine-specific AEs such as ‘Afluria-associated pain AE’ and generate corresponding OVAE terms. OVAE represents the VAE occurrences at specified age groups for different vaccines as reported in the package inserts. For example, according to the FDA-approved influenza vaccine FluMist package insert document [41], the most common adverse events in ≥ 10% FluMist recipients were runny nose or nasal congestion (ages 2–49 years), fever over 100°F (children ages 2–6 years), and sore throat (adults ages 18–49 years). Vaccinees under different age groups tend to have different occurrence rates for specific AEs. For example, the pain adverse event associated with influenza vaccine Afluria is > =60% for children 5–17 years of age, > = 40% for adults 18–64 years of age, and > =10% for adults 65 years of age and older [41]. Such information is now clearly represented using ontological axioms in OVAE. Therefore, the OVAE itself serves as a vaccine adverse event knowledge base. By querying the OVAE knowledge base, different scientific questions can be addressed. For example, OVAE was used to identify the top 10 vaccines according to numbers of asserted VAEs and the top 10 VAEs most frequently observed among vaccines. Such a system can be used in combination with VAERS to study VAEs systematically.

Such systematic retrieval and analysis of VAE results recorded in the licensed vaccine package insert documents could not have been performed without an ontology-based approach. The use of OAE thus has advanced the understanding of VAEs associated with licensed human vaccines.

#### (3) Analysis of genetic susceptibility to vaccine adverse events

Adverse events following vaccination, also called vaccine adverse event, were observed in some groups of people but not in others. This phenomenon is due to variations of the genetic factors altering individual’s susceptibility to vaccine adverse events. Recently an Ontology of Genetic Susceptibility Factors (OGSF) was applied and extended to model genetic susceptibility and genetic susceptibility factors associated with to vaccine adverse events [37]. A genetic susceptibility factor is a material basis of some genetic susceptibility. The OGSF ‘genetic susceptibility to vaccine adverse event’ , a subclass of OGSF ‘genetic susceptibility’ , is *realized in* an OAE_0000004:*vaccine adverse event* process. The genetic susceptibility factor exists as a part of a human vaccinee genome. The human genetic susceptibility factor will become a key participant in the vaccine adverse event process. Two use cases were studied: one relating to the human gene allele DBR1*15:01 as a genetic susceptibility factor that has been found to be a cause of multiple sclerosis in association with the influenza vaccine Pandemrix [42]; the other analyzing genetic polymorphisms associated with smallpox vaccine adverse events [43]. The OGSF modeling of these VAE specific cases requires the importing of many adverse event terms from OAE [37]. The combination of OGSF and OAE provides an effective way to represent and analyze the fundamental genetic mechanisms related with vaccine adverse events in some human populations with specific genetic characteristics.

### From vaccine-based AE analysis to drug-based AE analysis

Vaccine is a type of biological drug. Vaccines differ greatly from chemical drugs in many aspects. Firstly, vaccines are prepared from killed or live attenuated microbial organisms or large molecules (*e.g.*, recombinant proteins). Chemical drugs, in contrast, are compounds of small molecules. Host responses to vaccines and chemical drugs may therefore differ dramatically. Secondly, information relating to dose, time, and frequency is generally known precisely for vaccine administration, but is for drug administration often difficult to acquire. Thirdly, vaccines are mostly a preventive measure administered to healthy persons for disease prevention. In contrast, although preventive drugs exist, most drugs are given to patients under a pre-existing condition of illness, and response to drug treatment can be affected by both disease pathology and disease progression.

There are differences, too, on the side of AE monitoring. In the USA, vaccine AEs are monitored by the VAERS while drug AEs are monitored by the FAERS system. And for the reasons mentioned above, VAERS data on vaccine AEs are less noisy compared to the corresponding drug AE data in FAERS. Temporal association of an AE with a vaccination administration is much easier to detect than in the case of drug administration. Because vaccine recipients will typically be in a state of health with no other illnesses or complications prior to vaccination, the occurrence of undesirable symptoms can easily be hypothesized to have been induced by vaccination. As OAE applications in the vaccine domain have begun to yield useful interpretable results, the next step is to push forward the OAE applications in the more complex domain of drug AE analysis.

Several OAE-based drug AE analysis projects are in the implementation phase. For example, based on published drug package insert documents, OAE has been used to represent and analyse neuropathy adverse events induced by all possible chemical drugs [44]. This work is being refined and conducted to analyse the mechanisms of drug-induced neuropathy in patients. The FDA has been interested in applying ontology-based systems pharmacology to better analyse adverse drug reaction mechanisms and predict drug toxicology [45,46]. Currently, the FDA is investigating the usage of OAE as an adverse event data infrastructure to lay a foundation for a mechanistic-based systems pharmacology study of drug-induced cardiovascular toxicity [47,48]. In this case, OAE is being used as the bridge between MedDRA terms and relevant biological processes [47]. In addition, OAE is being used for literature mining of drug-drug and drug-molecule interactions [15]. An integration of OAE-based FAERS data analysis and literature mining has allowed the retrieval of gene interaction networks associated with one or a group of drug(s) and/or adverse events [48].

## Discussion and conclusions

The major contributions of this manuscript includes: (1) For the first time, we have formally announced and systematically described the new OAE ontology and how it extends and differs from the previous AEO [12]. The improved definition of ‘adverse event’ in current OAE makes a major framework change comparing to the previous (AEO) version of our ontology (Figures 1). The OAE term ‘causal adverse event’ now replaces the previous ‘adverse event’ definition. The latter term is to be used to represent individual adverse events known to be caused by medical interventions – as in the case of a swelling and redness of the skin at the injection site immediately after a flu shot. This change allows OAE to represent adverse events that are potentially not causal. This is aligned with the treatment of ‘adverse event’ in current clinical adverse event reporting systems, such as VAERS and FAERS. By this change, OAE extends its capability of representing reported adverse events. (2) We have now proposed the design patterns of adverse events and causal adverse events (Figures 1 and 2). These design patterns, together with ontological structure (Figure 3), provide a framework for systematic representation and analysis of adverse events and the factors affecting the adverse events. (3) We demonstrate that the OWL-based OAE ontology supports asserted and inferred hierarchy and reasoning (Figure 4). (4) Many new OAE terms have been added (Table 1). We have selectively introduced many branches of new terms, including those terms associated with causality assessments and different time regions and processes. (5) Furthermore, this article summarizes various works that represent many research contributions made with the support of OAE. Overall, OAE provides a unified and machine-readable ontological platform for representation and analysis of various adverse events and related issues (*e.g.*, causality assessment).

We are investigating or linking OAE with other related ontologies and knowledge bases that is designed to support a better understanding of complex adverse event processes. VAERS and FAERS mandate the usage of MedDRA as a controlled adverse event dictionary. MedDRA cannot be used to organize multiple levels of classification except through detailed knowledge provided by the users and the construction of complex queries [49]. While MedDRA cannot be ignored, an alignment between MedDRA and OAE will make it possible to leverage the OAE ontological data structure and the computational capability to utilize MedDRA data. The development of OAE is aligned with many existing ontologies such as Vaccine (VO) and Infectious Disease (IDO) Ontologies. The integration of OAE with VO has resulted in the generation of the Ontology of Vaccine Adverse Events (OVAE). OAE can also be co-studied with the Gene Ontology (GO), the Chemical Entities of Biological Interest (ChEBI), and published gene expression data resources to expand the networked adverse event data to genetic and chemical information resources.

The evidences described in this paper or other previous papers have shown that OAE works in adverse event representation and analysis. In the OAE-based comparative analysis of adverse events associated with trivalent (killed) inactivated influenza vaccine (TIV) and trivalent live attenuated influenza vaccine (LAIV) [8], we have also provided a side-by-side comparison on how OAE, MedDRA, and SNOMED classified the TIV and LAIV-associated vaccine adverse events. The comparative results were demonstrated in three supplemental figures of the published paper, with their titles: “Classification of TIV- and LAIV-enriched vaccine adverse events using OAE”, “Classification of TIV- and LAIV-enriched vaccine adverse events using MedDRA”, and “Classification of TIV- and LAIV-enriched vaccine adverse events using SNOMED-CT” [8]. The Discussion section of that paper have also described and discussed the comparative results [8]. This empirical evidence suggests that OAE has clear advantages over MedDRA and SNOMED in terms of adverse event classification. The descriptions in current manuscript provide more theoretical arguments. However, more empirical evidences would be needed to provide stronger arguments on the possible superiority of OAE over the alternatives.

Based on the evidences and theoretic arguments described in this paper, we contend that OAE provides a novel and powerful framework for analyzing possible causal associations between medical interventions and adverse events and the underlying mechanisms. The integration of OAE with other applications such as literature mining makes it possible to systemically analyze molecular mechanisms of adverse events. For example, OAE is being used for literature mining of gene interaction networks related to fever vaccine adverse events [14]. The OAE can also be integrated with statistical analysis of AE case report data [8] and potentially with high throughput gene expression data analysis for better understanding fundamental gene interactions and pathways of various adverse events. Such studies will likely impact our ability to diagnose, preventing, and treat adverse events in the future.

### Availability and requirements

The OAE project site is: http://www.oae-ontology.org. OAE is listed in the OBO Foundry library (http://www.obofoundry.org/). It is also available in the NCBO BioPortal (http://bioportal.bioontology.org/) and in Ontobee (http://www.ontobee.org) for public visualization and querying. The source code of the ontology is freely available under the Apache License 2.0.

## Abbreviations

AEO: Adverse event ontology; AERO: Adverse event reporting ontology; BFO: Basic formal ontology; BSPO: Spatial ontology; CDISC: Clinical data interchange consortium; CODAE: Ontology-based detection of adverse events; CTCAE: Common terminology criteria for adverse events; DOID: Disease ontology; FAERS: FDA adverse events reporting system; FDA: Food and Drug Administration (FDA); FMA: Foundational Model of Anatomy; IAO: Information artifact ontology; IDO: Infectious disease ontology; MedDRA: Medical dictionary for regulatory activities; OAE: Ontology of adverse events; OBI: Ontology for biomedical investigations; OBO: Open biomedical/biological ontologies; OGMS: Ontology for general medical science; PATO: Phenotypic quality ontology; PRR: Proportional reporting ratio; RO: Relation ontology; TIV: Trivalent inactivated influenza vaccine; LAIV: Live attenuated influenza vaccine; UBERON: Uber anatomy ontology; VAERS: Vaccine adverse event; VO: Vaccine ontology; WHO-ART: WHO’s adverse reaction terminology.

## Competing interests

The authors declare no competing interests.

## Authors’ contributions

YH initiated and leads the development of the AEO and OAE as the primary developer. SS led the OAE case study on influenza vaccine AE analysis and has started the OAE application in drug adverse event studies. ZX provided technical support at the early stages of AEO/OAE development. AG contributed modeling of neuropathy adverse events induced by chemical drugs. SZ and DJ contributed to the addition of many AE terms to OAE under the mentoring of YH and SS. YL suggested new OAE terms and has applied OAE to vaccine adverse event susceptibility modeling. LT guided the OAE modeling of drug adverse events in the domain of pharmacovigilance. CT contributed time modeling analysis of vaccine adverse event. BS contributed logical elements, including ensuring that OAE aligns with BFO. All co-authors participated in writing, reviewing, discussion, and editing of the manuscript. All authors read and approved the final manuscript.

## Supplementary Material

Additional file 1: Table S1 Ontology classes used in the manuscript. Click here for file

Additional file 2: Table S2 Ontology relations used in the manuscript. Click here for file

## References

[B1] Scheuermann RH Ceusters W Smith B Toward an Ontological Treatment of Disease and Diagnosis Proceedings of the 2009 AMIA Summit on Translational Bioinformatics 2009 116 120 PMC3041577 21347182

[B2] Varricchio F Iskander J Destefano F Ball R Pless R Braun MM Chen RT Understanding vaccine safety information from the Vaccine Adverse Event Reporting System Pediatr Infect Dis J 2004 23 4 287 294 15071280 10.1097/00006454-200404000-00002

[B3] FDA U FDA Adverse Event Reporting System (FAERS) (formerly AERS) 2013 URL: http://www.fda.gov/Drugs/GuidanceComplianceRegulatoryInformation/Surveillance/AdverseDrugEffects/default.htm, accessed on June 21, 2013

[B4] Brown EG Wood L Wood S The medical dictionary for regulatory activities (MedDRA) Drug Saf 1999 20 2 109 117 10082069 10.2165/00002018-199920020-00002

[B5] NCI Common Terminology Criteria for Adverse Events (CTCAE) http://ctep.cancer.gov/reporting/ctc.html. Accessed on May 15, 2013

[B6] WHO’s Adverse Reaction Terminology (WHO-ART) http://www.nlm.nih.gov/research/umls/sourcereleasedocs/current/WHO/. Accessed on March 17, 2013

[B7] Brown EG Methods and pitfalls in searching drug safety databases utilising the Medical Dictionary for Regulatory Activities (MedDRA) Drug Saf 2003 26 3 145 158 12580645 10.2165/00002018-200326030-00002

[B8] Sarntivijai S Xiang Z Shedden KA Markel H Omenn GS Athey BD He Y Ontology-based combinatorial comparative analysis of adverse events associated with killed and live influenza vaccines PLoS One 2012 7 11 e49941 23209624 10.1371/journal.pone.0049941 PMC3509157

[B9] He Y Cowell L Diehl AD Mobley HL Peters B Ruttenberg A Scheuermann RH Brinkman RR Courtot M Mungall C Xiang Z Chen F Todd T Colby LA Rush H Whetzel T Musen MA Athey BD Omenn GS Smith B VO: Vaccine Ontology The 1st International Conference on Biomedical Ontology (ICBO-2009): July 24–26 2009 2009 Buffalo, NY, USA: Nature Precedings http://precedings.nature.com/documents/3552/version/1

[B10] Lin Y He Y Ontology representation and analysis of vaccine formulation and administration and their effects on vaccine immune responses J Biomed Semantics 2012 3 1 17 23256535 10.1186/2041-1480-3-17 PMC3639077

[B11] Ceusters W Capolupo M de Moor G Devlies J Smith B An evolutionary approach to realism-based adverse event representations Methods Inf Med 2011 50 1 62 73 21057717 10.3414/ME10-02-0016 PMC3103706

[B12] He Y Xiang Z Sarntivijai S Toldo L Ceusters W AEO: a realism-based biomedical ontology for the representation of adverse events Adverse Event Representation Workshop, International Conference on Biomedical Ontologies (ICBO-2011): July 26–30 2011 2011 Buffalo, NY, USA: CEUR Workshop Proceedings 309 315 http://ceur-ws.org/Vol-833/paper359.pdf

[B13] Courtot M Brinkman RR Ruttenberg A The logic of surveillance guidelines: an analysis of vaccine adverse event reports from an ontological perspective PLoS One 2014 9 3 e92632 24667848 10.1371/journal.pone.0092632 PMC3965435

[B14] Hur J Ozgur A Xiang Z He Y Identification of fever and vaccine-associated gene interaction networks using ontology-based literature mining J Biomed Semantics 2012 3 1 18 23256563 10.1186/2041-1480-3-18 PMC3599673

[B15] Gurulingappa H Mateen-Rajput A Toldo L Extraction of potential adverse drug events from medical case reports J Biomed Semantics 2012 3 1 15 23256479 10.1186/2041-1480-3-15 PMC3599676

[B16] Gurulingappa H Rajput AM Roberts A Fluck J Hofmann-Apitius M Toldo L Development of a benchmark corpus to support the automatic extraction of drug-related adverse effects from medical case reports J Biomed Inform 2012 45 5 885 892 22554702 10.1016/j.jbi.2012.04.008

[B17] Gurulingappa H Toldo L Rajput AM Kors JA Taweel A Tayrouz Y Automatic detection of adverse events to predict drug label changes using text and data mining techniques Pharmacoepidemiol Drug Saf 2013 22 11 1189 1194 23935003 10.1002/pds.3493

[B18] Smith B Ashburner M Rosse C Bard J Bug W Ceusters W Goldberg LJ Eilbeck K Ireland A Mungall CJ Leontis N Rocca-Serra P Ruttenberg A Sansone SA Scheuermann RH Shah N Whetzel PL Lewis S The OBO Foundry: coordinated evolution of ontologies to support biomedical data integration Nat Biotechnol 2007 25 11 1251 1255 17989687 10.1038/nbt1346 PMC2814061

[B19] Courtot M Goldfain A He Y Ruttenberg A Adverse Event Representation Workshop International Conference on Biomedical Ontologies 2011 (ICBO 2011) 2011; University at Buffalo, NY 2011 http://icbo.buffalo.edu/2011/workshop/adverse-events

[B20] He Y Toldo L Burns G Tao C Abernethy DR A 2012 Workshop: Vaccine and Drug Ontology in the Study of Mechanism and Effect (VDOSME 2012) J Biomed Semantics 2012 3 1 12 23249650 10.1186/2041-1480-3-12 PMC3554582

[B21] Tao C He Y Arabandi S A 2013 Workshop: Vaccine and Drug Ontology Studies (VDOS 2013) J Biomed Semantics 2014 5 1 16 24650607 10.1186/2041-1480-5-16 PMC3994568

[B22] Zhou W Pool V Iskander JK English-Bullard R Ball R Wise RP Haber P Pless RP Mootrey G Ellenberg SS Braun MM Chen RT Surveillance for safety after immunization: Vaccine Adverse Event Reporting System (VAERS)–United States, 1991–2001 MMWR Surveill Summ 2003 52 1 1 24 12825543

[B23] Burnstead B Furlan G Unifying drug safety and clinical databases Curr Drug Saf 2013 8 1 56 62 23656448 10.2174/1574886311308010008

[B24] Smith B Ceusters W Ontological realism: A methodology for coordinated evolution of scientific ontologies Appl Ontol 2010 5 139 188 21637730 10.3233/AO-2010-0079 PMC3104413

[B25] Xiang Z Courtot M Brinkman RR Ruttenberg A He Y OntoFox: web-based support for ontology reuse BMC Res Notes 2010 3 175 20569493 10.1186/1756-0500-3-175 PMC2911465

[B26] Grenon P Smith B SNAP and SPAN: Towards Dynamic Spatial Ontology Spat Cogn Comput 2004 4 1 69 103

[B27] MacKenzie SH Clark AC Death by caspase dimerization Adv Exp Med Biol 2012 747 55 73 22949111 10.1007/978-1-4614-3229-6_4 PMC3877935

[B28] Marcos E Zhao B He Y The Ontology of Vaccine Adverse Events (OVAE) and its usage in representing and analyzing adverse events associated with US-licensed human vaccines J Biomed Semantics 2013 4 40 24279920 10.1186/2041-1480-4-40 PMC4177204

[B29] Mungall CJ Torniai C Gkoutos GV Lewis SE Haendel MA Uberon, an integrative multi-species anatomy ontology Genome Biol 2012 13 1 R5 22293552 10.1186/gb-2012-13-1-r5 PMC3334586

[B30] Rosse C Mejino JL Jr A reference ontology for biomedical informatics: the Foundational Model of Anatomy J Biomed Inform 2003 36 6 478 500 14759820 10.1016/j.jbi.2003.11.007

[B31] NCBI Taxonomy ontology http://www.obofoundry.org/cgi-bin/detail.cgi?id=ncbi_taxonomy. Accessed on March 17, 2013

[B32] Naranjo CA Busto U Sellers EM Sandor P Ruiz I Roberts EA Janecek E Domecq C Greenblatt DJ A method for estimating the probability of adverse drug reactions Clin Pharmacol Ther 1981 30 2 239 245 7249508 10.1038/clpt.1981.154

[B33] Evans SJ Waller PC Davis S Use of proportional reporting ratios (PRRs) for signal generation from spontaneous adverse drug reaction reports Pharmacoepidemiol Drug Saf 2001 10 6 483 486 11828828 10.1002/pds.677

[B34] Heaney RP Kopecky S Maki KC Hathcock J Mackay D Wallace TC A review of calcium supplements and cardiovascular disease risk Advances in nutrition 2012 3 6 763 771 23153730 10.3945/an.112.002899 PMC3648700

[B35] Pearl J Causality (2nd edition) 2009 Cambridge University Press

[B36] Tao C He Y Yang H Gregory PA Chute CG Ontology-based time information representation of vaccine adverse events in VAERS for temporal analysis J Biomed Semantics 2012 3 1 13 23256916 10.1186/2041-1480-3-13 PMC3554604

[B37] Lin Y He Y The Ontology of Genetic Susceptibility Factors (OGSF) and its application in modeling genetic susceptibility to vaccine adverse events J Biomed Semantics 2014 5 19 24963371 10.1186/2041-1480-5-19 PMC4068904

[B38] The protege ontology editor http://protege.stanford.edu/

[B39] Tao C Wei WQ Solbrig HR Savova G Chute CG CNTRO: A Semantic Web Ontology for Temporal Relation Inferencing in Clinical Narratives AMIA Annu Symp Proc 2010 2010 787 791 21347086 PMC3041418

[B40] U.S. Food and Drug Administration Vaccines Licensed for Immunization and Distribution in the US with Supporting Documents URL: http://www.fda.gov/BiologicsBloodVaccines/Vaccines/ApprovedProducts/UCM093830.htm, accessed on April 3, 2013

[B41] US FDA Afluria package insert information URL: http://www.fda.gov/downloads/BiologicsBloodVaccines/Vaccines/ApprovedProducts/UCM263239.pdf. Accessed on April 18, 2014

[B42] Vrethem M Malmgren K Lindh J A patient with both narcolepsy and multiple sclerosis in association with Pandemrix vaccination J Neurol Sci 2012 321 1–2 89 91 22841884 10.1016/j.jns.2012.07.025

[B43] Reif DM McKinney BA Motsinger AA Chanock SJ Edwards KM Rock MT Moore JH Crowe JE Genetic basis for adverse events after smallpox vaccination J Infect Dis 2008 198 1 16 22 18454680 10.1086/588670 PMC2746083

[B44] He Y Updates on the development of the Ontology of Adverse Events (OAE) and its applications The 2012 Vaccine and Drug Oontology in the Study of Mechanism and Effect (VDOSME) workshop 2012 Graz, Austria http://kr-med.org/icbofois2012/vdosme/docs/presentations/OAE_VDOSME2012_He.pdf

[B45] Abernethy DR Woodcock J Lesko LJ Pharmacological mechanism-based drug safety assessment and prediction Clin Pharmacol Ther 2011 89 6 793 797 21490594 10.1038/clpt.2011.55

[B46] Bai JP Abernethy DR Systems pharmacology to predict drug toxicity: integration across levels of biological organization Annu Rev Pharmacol Toxicol 2013 53 451 473 23140241 10.1146/annurev-pharmtox-011112-140248

[B47] Sarntivijai S Lin Y Blair E Burkhart KK He Y Omenn GS Athey BD Abernethy DR The ontology representation of adverse events with composite symptoms: expanding Ontology of Adverse Events to describe drug-induced cardiotoxicity American Society for Clinical Pharmacology and Therapeutics 2014 Annual Meeting (ASCPT-2014) 2014 Atlanta, Georgia http://www.ascpt.org/Portals/8/docs/Meetings/2014%2020Annual%2020Meeting/Speaker%2020Presentations/Thursday/TKI_Sirarat%2020Sarntivijai.pdf

[B48] Sarntivijai S Hur J Ozgur A Burkhart KK He Y Omenn GS Athey BD Abernethy DR Predicting gene interactions of tyrosine kinase inhibitor-induced cardiotoxicity with ontology of adverse events-assisted bioinformatics American Society for Clinical Pharmacology and Therapeutics 2014 Annual Meeting (ASCPT-2014) 2014 Atlanta, Georgia

[B49] Bousquet C Henegar C Louet AL Degoulet P Jaulent MC Implementation of automated signal generation in pharmacovigilance using a knowledge-based approach Int J Med Inform 2005 74 7–8 563 571 15955732 10.1016/j.ijmedinf.2005.04.006

