# Supplementary material for: OAE: The Ontology of Adverse Events
Source: J Biomed Semantics. 2014 Jul 5;5:29. doi: 10.1186/2041-1480-5-29 (PMC4120740; doi:10.1186/2041-1480-5-29)
Supplement: Additional file 1: Table S1 — Ontology classes used in the manuscript. [file 2041-1480-5-29-S1.doc]

**Additional file 1: Table S1. Ontology classes used in the manuscript**

| # | Classes | Sources and term IDs | Parent class | Figures |
| --- | --- | --- | --- | --- |
| 1 | planned process | http://purl.obolibrary.org/obo/OBI_0000011 | process (BFO) | 1, 2, 3 |
| 2 | process | http://purl.obolibrary.org/obo/BFO_0000007 | occurrent (BFO) | 1, 2, 3 |
| 3 | pathological bodily process | http://purl.obolibrary.org/obo/OGMS_0000061 | bodily process (OGMS) | 1, 2, 3 |
| 4 | medical intervention | http://purl.obolibrary.org/obo/OAE_0000002 | planned process (OBI) | 1, 2, 3 |
| 5 | adverse event | http://purl.obolibrary.org/obo/OAE_0000001 | pathological bodily process (OGMS) | 1, 3 |
| 6 | time at medical intervention | http://purl.obolibrary.org/obo/OAE_0000071 | zero-dimensional temporal region (BFO) | 1, 3 |
| 7 | causal adverse event | http://purl.obolibrary.org/obo/OAE_0000003 | adverse event (OAE) | 1, 2,3 |
| 8 | adverse event time period | http://purl.obolibrary.org/obo/OAE_0001816 | one-dimensional temporal region (BFO) | 1, 3 |
| 9 | patient | http://purl.obolibrary.org/obo/OAE_0001817 | organism (NCBITaxon) | 1, 2, 3 |
| 10 | bodily process | http://purl.obolibrary.org/obo/OGMS_0000060 | process (BFO) | 2, 3 |
| 11 | time at medical intervention | http://purl.obolibrary.org/obo/OAE_0000071 | zero-dimensional temporal region | 2 |
| 12 | anatomical entity | http://purl.obolibrary.org/obo/UBERON_0001062 | material entity (BFO) | 2, 3 |
| 13 | initial stage sub-process after medical intervention | http://purl.obolibrary.org/obo/OAE_0001813 | pathological bodily process (OGMS) | 2 |
| 14 | intermediate stage causal AE process | http://purl.obolibrary.org/obo/OAE_0001814 | pathological bodily process (OGMS) | 2 |
| 15 | late stage AE formation sub-process | http://purl.obolibrary.org/obo/OAE_0001815 | pathological bodily process (OGMS) | 2 |
| 16 | time interval for initial stage sub-process after medical intervention | http://purl.obolibrary.org/obo/OAE_0001810 | one-dimensional temporal region (BFO) | 2 |
| 17 | time interval for intermediate stage causal AE sub-process | http://purl.obolibrary.org/obo/OAE_0001811 | one-dimensional temporal region (BFO) | 2 |
| 18 | time interval for last stage AE formation sub-process | http://purl.obolibrary.org/obo/OAE_0001812 | one-dimensional temporal region (BFO) | 2 |
| 19 | time interval for causal AE | http://purl.obolibrary.org/obo/OAE_0001818 | adverse event time period | 2 |
| 20 | entity | http://purl.obolibrary.org/obo/BFO_0000001 | Thing | 3 |
| 21 | continuant | http://purl.obolibrary.org/obo/BFO_0000002 | entity (BFO) | 3 |
| 22 | occurrent | http://purl.obolibrary.org/obo/BFO_0000003 | entity (BFO) | 3 |
| 23 | information content entity | http://purl.obolibrary.org/obo/IAO_0000030 | generically dependent continuant (BFO) | 3 |
| 24 | material entity | http://purl.obolibrary.org/obo/BFO_0000040 | Independent continuant (BFO) | 3 |
| 25 | temporal region | http://purl.obolibrary.org/obo/BFO_0000008 | occurrent (BFO) | 3 |
| 26 | adverse event causality score | http://purl.obolibrary.org/obo/OAE_0001022 | data item (IAO) | 3 |
| 27 | Naranjo ADR Probability score | http://purl.obolibrary.org/obo/OAE_0000160 | adverse event causality score (OAE) | 3 |
| 28 | causal adverse event hypothesis | http://purl.obolibrary.org/obo/OAE_0000028 | hypothesis textual entity (IAO) | 3 |
| 29 | organism | http://purl.obolibrary.org/obo/OBI_0100026 | material entity (BFO) | 3 |
| 30 | eye adverse event | http://purl.obolibrary.org/obo/OAE_0000443 | adverse event (OAE) | 3 |
| 31 | severe adverse event | http://purl.obolibrary.org/obo/OAE_0000631 | adverse event (OAE) | 3 |
| 32 | vaccine adverse event | http://purl.obolibrary.org/obo/OAE_0000004 | adverse event (OAE) | 3 |
| 33 | vaccination | http://purl.obolibrary.org/obo/VO_0000002 | medical intervention (OAE) | 3 |
| 34 | drug administration | http://purl.obolibrary.org/obo/OAE_0000011 | medical intervention (OAE) | 3 |
