# Supplementary material for: OAE: The Ontology of Adverse Events
Source: J Biomed Semantics. 2014 Jul 5;5:29. doi: 10.1186/2041-1480-5-29 (PMC4120740; doi:10.1186/2041-1480-5-29)
Supplement: Additional file 2: Table S2 — Ontology relations used in the manuscript. [file 2041-1480-5-29-S2.doc]

**Additional file 2: Table S2. Ontology relations used in the manuscript:**

| # | Property terms | Sources and term IDs | Figures |
| --- | --- | --- | --- |
| 1 | has participant | http://www.obofoundry.org/ro/ro.owl#has_participant | 1, 2, 3 |
| 2 | exists at | http://purl.obolibrary.org/obo/BFO_0000108 | 1, 2 |
| 3 | preceded by | http://purl.obolibrary.org/obo/BFO_0000062 | 1, 2, 3 |
| 4 | induced by | http://purl.obolibrary.org/obo/OAE_0000025 | 1, 2 |
| 5 | part_of | http://www.obofoundry.org/ro/ro.owl#part_of | 2 |
